# Comparing Pears to Apples: Unlike Dogs, Cats Need Habituation before Lab Tests

**DOI:** 10.3390/ani12213046

**Published:** 2022-11-06

**Authors:** Stefania Uccheddu, Ádám Miklósi, Sarolt Gintner, Márta Gácsi

**Affiliations:** 1 MTA-ELTE Comparative Ethology Research Group, 1117 Budapest, Hungary; 2 Department of Ethology, Eötvös Loránd University, 1117 Budapest, Hungary

**Keywords:** family cat, dog, comparative ethology, testability

## Abstract

**Simple Summary:**

Comparative studies can help us better understand our family pets’ social and cognitive behaviours and gain more insights in the evolution of some human abilities. However, the comparison of the behaviours of cats and dogs in a standard laboratory environment is not without challenges. Though recently they play a similar role in modern Western societies as pets, both their evolutionary history and individual experiences are different. We tested the spontaneous behaviour of companion cats and dogs in the same novel laboratory environment, and if needed, we tried to habituate them in the presence of their owners and an unfamiliar experimenter. To pass the habituation test, subjects were expected to play with the experimenter or accept food from them. All dogs passed the test on the first occasion, while almost 60% of the cats needed habituation and some could not reach the criteria even after three habituation sessions. More experienced cats (which had the opportunity to meet strangers and explore unfamiliar places) were not more successful, in fact, younger cats passed more easily. We found marked differences between dogs and cats in all behavioural variables; compared to dogs, cats spent more time crouching and close to their box, while less time exploring or close to the owner. Our findings are important not only regarding the test methods of cats and the interpretation of their data collected in the laboratory so far but, in a more general sense, on developing future comparative experiments.

**Abstract:**

Research on the socio-cognitive skills of different species often benefit from comparative experiments, however, the ecology of the species and development of the individuals may differently determine how they react to the same test situation. In this study, our aims were twofold: to observe and compare the spontaneous behaviour of companion cats and dogs in the same novel environment, and to habituate them (if needed) to the novel environment in the presence of their owners and an unfamiliar experimenter. The behaviour of 62 family cats, 31 experienced (which had the opportunity to meet strangers and explore unfamiliar places) and 31 inexperienced cats, and 27 family dogs was compared in an unfamiliar room. The subjects’ behaviour was coded during the first five minutes in the presence of two passive persons, their owners, and an unfamiliar experimenter. Then, based on a set of rules, first the owner, and then the experimenter tried to initiate interactions with the subjects and the subjects’ willingness to interact was evaluated. To pass the habituation test, subjects were expected to play with the experimenter or accept food from them. All dogs passed the test on the first occasion, while almost 60% of the cats failed. The cats’ experience did not play a significant role, in fact, younger cats passed more easily. We found marked differences between dogs and cats in all behavioural variables; compared to dogs, cats spent more time crouching and close to their box, while less time exploring or close to the owner. We did not find a difference in the cats’ behaviour based on their experience. Our results support the hypothesis that unlike dogs, cats need extensive habituation in a novel environment. This could partially be explained by the difference in the ecology and/or domestication process of the species, although developmental effects cannot be excluded. Our findings have fundamental consequences not only for the considerations of the testability of cats and on the interpretation of their data collected in the laboratory, but in a more general sense on developing comparative experiments.

## 1. Introduction

Dispersal and/or migration to a novel environment poses significant risk to any animal because of the potential dangers and the separation from its well-known home range [1]. Not all species show such tendencies, but if they do, moving to a novel environment takes place during specific seasons or at a specific age [2]. Thus, the ecology of the species determines how individuals react to sudden or gradual changes in their natural environment.

Introducing an animal to a novel place has been used as a laboratory experimental paradigm to investigate exploratory activity and fear responses [3]. In these, so called ‘open field’ tests, individuals can choose between different strategies; they either move around actively and collect information about the novel place (‘exploration’) or they show passive (‘freezing’) or active (‘escape’) avoidance. The novel environment may range from a simple empty open area to a stimulus enriched environment (i.e., with objects and/or social partners) [4].

Rodents have been the most popular subjects for the open-field test to measure exploratory behaviour [5]. Typically, the amount of activity is interpreted as reflecting the reaction to novelty in response to continued or repeated exposure to a novel environment [6]. The open field test has been applied to a wide range of animals including farm animals such as ruminants, pigs, horses and poultry [4], and the exploratory behaviour of companion animals such as dogs has also been investigated [7]. 

However, sudden exposure to a novel situation is more likely to elicit passive behaviour accompanied by fear. Thus, the same test was also used to detect the subjects’ tendency to show withdrawal in such a context. Studies on emotionality in rats revealed that initial fear decreased if the subjects had some experience with similar situations [8].

Comparative studies are essential to determine the origins and different degrees of certain social and cognitive abilities. However, it is sometimes difficult to balance the contradictory requirements of a valid comparison such as whether to assess the abilities of each species using the same standard method (location, paradigm) or adapt the conditions to the needs of a given species.

Our main aim was to directly compare the behavioural responses of companion dogs and cats to a novel environment (laboratory room) as so far, most socio-cognitive tests on cats have been carried out at home (e.g., [9,10,11]) or in a familiar place [12], as cats were reported to be sensitive to changes in the environment [13,14].

In these two species, evolutionary and ecological ultimate factors may include the use of home range in the ancestors, the social system, the history of domestication, and the role and historic function of the interaction with humans. The developmental (e.g., socialisation) experience, the relationship with the owner, and general experience with a novel environment and people may prove to be important as proximate factors.

It is generally assumed that the establishment of territory is more significant for cats than dogs, and there is no overlap of home ranges between adjacent feral cats (see for example [15]). Feral cats may be flexible in patrolling a home range or defend a territory if food, shelter, and mate resources are available [16,17]. In contrast, feral dogs may have partly overlapping home ranges, and may get into conflict only when they meet at feeding places [18]. Thus, behaviours in a novel environment may reflect different ecological and coping strategies in cats, which may make them more sensitive to a change in environment.

Companion cats are more and more often involved in scientific experiments (e.g., [19]), and it is crucial to understand whether the laboratory test location used for other species (e.g., dogs, pigs) is similarly viable for them. Comparative research on the socio-cognitive abilities of dogs and cats (e.g., [9,20]) relies on the fact that both species were domesticated many thousand years ago [21,22] and are nowadays kept as companions. However, there are important differences in their domestication processes and keeping conditions. In dogs, domestication contributed significantly to both the emergence of attachment behaviour towards humans, for example, using the owner as a safe haven [23] and a human-compatible social competence [24], so that, as stated in [25], “the most significant compound of the environment for many dogs is represented by the presence of the owner”. In contrast, historically, feral cats were only allowed in human proximity to keep the living environment free from unwelcomed small animals [26], without the need for displaying dependency or human analogue social traits [27]. Since primordial human––cat relationships were based only on mere utility [28], as a consequence of no selection for other features, the owner may not play such a central role in the social relationship of cats [29]. Thus, even if companion cats living with their owners occupy a similar ecological niche to that of the dog and they share the same human environment and routine with similar interspecific social stimuli (humans), the relationship between cats and their caregivers does not seem to be characterised by the security providing role of the owner. Dogs tend to interact with their owners as a “social unit” [30], however, family cats are predominantly kept for the purpose of providing companionship for their human owner [31], even if cat owners described the relationship as resembling the dog-owner relationship [10]. Moreover, the interspecific bond between dogs and humans (social unit) is relevant not only inside, but also outside the home, unlike the cat–human bond.

When cats are exposed to an entirely novel situation, they tend to spend more time hiding or attempting to hide [32,33], lying and standing, freezing, crawling, startle, and retreat from unfamiliar humans [34]. Most likely based on these findings or on the experimenters’ personal experience, thus far in socio-cognitive studies, companion cats have mostly been tested at home, even though their testability had not been directly assessed in a laboratory environment [9,10,35]. Though Kraus and colleagues [20] compared dogs and cats in a two-way-choice test in their home environment, even in this familiar location, 42% of the cats dropped out due to struggling against handling or a loss of motivation (and all but one of the successfully tested cats had already participated in a previous experiment).

Of note, even if there are differences among countries due to cultural reasons, city cats are typically kept indoors (with little experience), whereas dogs are more used to getting around outside and to encounter a wide range of people, which can in turn affect the differences in experience. For well socialised family dogs, a typical novel environment should not be considered as very different from what they experience in their everyday life (e.g., visiting different places and environments with the owner; [25]). However, this is certainly not the case for cats that are only taken out of the apartment to the veterinary clinic. 

The main aim of the present study was to compare the behaviours of companion cats and dogs in the same novel environment in the presence of the owner and an unfamiliar experimenter. We considered that an individual habituated to the novel environment if they explored the unfamiliar environment and accepted food from or played with both the owner and the unfamiliar female experimenter, considering that such behaviours emerge when the level of fear is low [4]. We hypothesised that there would be a significant difference in both the observed behaviours and the success to pass (obtaining interaction with the experimenter) between cats and dogs. We expected that dogs would typically not need habituation to the laboratory environment, that is, they would pass the test quickly. However, in a novel environment, companion cats may show passive withdrawal and only gradually display some exploration behaviours. Moreover, within cats, we expected an effect of experience (having the opportunity to meet strangers and explore unfamiliar places), making more experienced cats easier to habituate. We predicted that compared to cats with a lot of experience about their surrounding environment (outdoor cats and indoor cats that regularly meet strangers), inexperienced cats would need repeated exposure to achieve similar levels of habituation and that a relatively large portion of these cats would not habituate, even after three exposures. 

Consequently, we expected that dogs would display active exploration (spending less time in hiding and crouching) and spend more time close to the owner than cats. The behaviour of experienced cats was expected to be more similar to that of dogs. As exploration of the environment and potential social partners are more important for young individuals, we expected that the cats’ age may have an effect on the speed of habituation. 

Of note, in this study, we wanted to compare the responses of typical family cats and family dogs in the same research environment, and our aim was not to determine the exact underlying reasons for any behavioural differences between them, mainly due to the several factors that vary between the two species (ecology, domestication process, and experience), it is not possible to conclude which drive these differences. 

## 2. Materials and Methods

### 2.1. Ethical Permission

All procedures of this study were approved by the Ethical Committee of Eötvös Loránd University (Permission # PE/EA/1005-5/2018) and conducted in accordance with the recommendations of the Hungarian State Health and Medical Service. Informed consent was obtained from all owners and they participated in the test with their pets on a voluntary basis. The owners were present during the tests, and they were told that they could terminate the experiment at any time if they thought their pet was being exposed to unwanted stress.

### 2.2. Subjects

Participants were recruited through advertisements online and on the University community notice boards. All dog and cat subjects were at least four months old, and had spent at least one month with the owner at the time of testing. No subject was kept exclusively in the garden.

We tested 62 cats (31 females, 55 cats were neutered), which did not belong to any breed (with the exception of one Siamese and one Maine coon). Their age was 3.8 ± 3.2 (mean ± SE) years, and most of them (N = 50) were kept inside exclusively. Based on the report of the owners (questionnaire), we categorised the cats as experienced (E) vs. inexperienced (IE) individuals depending on their opportunity to learn about and habituate to unfamiliar stimuli. Cats that could live both indoors and outdoors, or could meet strangers more than once per week, or had the opportunity to visit new places once per week were considered as E. According to these criteria, 50% of the cats were considered E.

We tested 27 dogs (16 females, 10 dogs were neutered), with ages of 4.8 ± 4.0 years (mean ± SD). To provide a valid comparison of the two species for a later test battery, we recruited a variety of relatively small sized dogs with a maximum 30 cm height at the withers and 15 kg weight. We tested 27 dogs from 15 breeds (four Yorkshire terriers, three Jack Russell terriers, two dachshunds, two Bolognese, two Havanese, one miniature dachshund, one papillon, one miniature pinscher, one Chinese crested dog, one French bulldog, one Maltese, one poodle, one West Highland white terrier, one fox terrier, and one beagle) and four mongrels. According to the criteria applied in the case of cats, all dogs were considered E.

The timing of the cats’ and dogs’ tests was based on the owners’ availability.

### 2.3. Experimental Setup

The cats and dogs were individually tested in an unfamiliar room in the laboratory of the Department of Ethology. The laboratory was cleaned after each test. The tests were video recorded using four fixed cameras. The owners were asked not to feed the animals in the two hours prior to the test.

**Figure 1:**
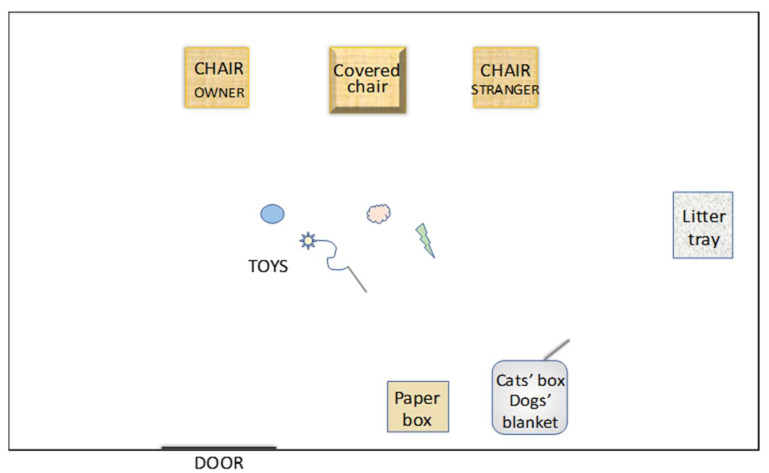
Illustration of the lab environment in the habituation test.

The observation room (3 × 5.4 m) was furnished with three chairs (positioned next to the wall at 50 cm from each other), a litter box, a carton box, and play objects (Figure 1). The three chairs were located at the wall, the opposite side of the entrance door. An empty chair covered by a cloth was placed between the chairs occupied by the owner and the experimenter, who was always a young female. Upon arrival, the owner and the experimenter entered the testing room with the subject. Cats were taken to the test area inside the box, dogs entered on leash (most dogs did not have a box). During the experiment, both the experimenter and the owner were present with the subject.

### 2.4. Procedure

**Figure 2:**
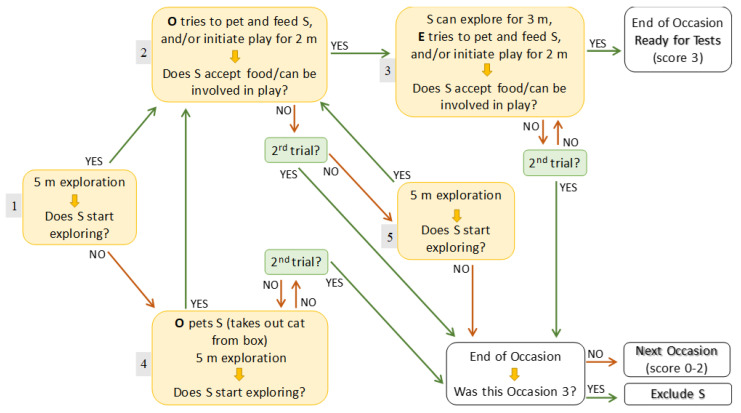
The flow–chart of the habituation/test procedure; owner’s (O) and experimenter’s (E) actions were based on the behaviour of the subject (S). The numbers in the small grey boxes on the left of the large (action) boxes help illustrate the steps of the potential routes of the chart. Steps 2, 3, and 4 could be tried twice (‘trials’). The subjects’ behaviour was analysed in detail only for the first 5 min (grey box 1) during the first occasion.

Depending on the behaviour of the subject, 1–3 habituation tests (occasions) were carried out (maximum 3 weeks elapsed between two test occasions). Each occasion was conducted following the same rules; for the detailed procedure and outcomes, see the flow chart presented in Figure 2. 

Cat owners were asked to leave the transport box close to the carton box, open the door, and sit down. Dog owners were asked to take the dog off leash after entering the room and sit down. In the case of dogs, instead of a box, a favourite blanket was placed close to the carton box. Subjects were not restricted in any way during the experimental procedure; they were free to explore the room and/or interact with the humans. During the first 5 min, the owner and the stranger sat passively on a chair so that the behaviour of the animals could be compared (‘open field’).

After 5 min elapsed, if the subject started to explore the room or approached the owner, the owner initiated interaction with it by calling, petting, playing, and/or offering food. During these simple situations, all owners interacted with their pets in basically the same way; they did not move much but rather encouraged the animal to approach them and take the offered food or play with the offered toys. If the owner could involve the pet in some play activity and/or the pet accepted the offered treat, the owner sat down, and the experimenter started to initiate interaction with the animal in the same way the owner had before. The experimenter measured the time, constantly evaluated the subject’s responses, and decided on the next step based on them. The habituation test could last <10 min with a subject who was keen to explore the environment and interact with the persons, going through the least number of steps (route 1–2–3; Figure 2).

If the cat did not leave the transport box after the first 5 min elapsed, the owner took off the top of the box, gently picked the cat up, and placed it beside the box. There was no dog who did not begin to explore, while cats could go back to the box during the entire test. Then, the procedure went on depending on the responses of the animal until either the subject passed the test, or the habituation process was terminated. In the latter case, the habituation continued in the next occasion (on a different day), if that was not the third (last) occasion. An occasion could last up to 29 min with subjects who went through the maximum number of steps (route 1–4–4–2–5–2–3–3; Figure 2). 

### 2.5. Data Analysis

Two types of behavioural analyses were carried out. First, based on the outcome of the test (see flow chart), a score was given to the subjects at the end of each occasion. Second, the behaviour of each subject during the first 5 min of the test (when the human partners were passive) was coded (Solomon Coder beta^®^ 15.01.13 copyright András Péter) and analysed separately. 

### 2.6. Scoring

Score 0 was assigned to animals that did not start exploring/leave the box (Figure 2; route 1–4–4). Score 1 was assigned to animals that showed some exploratory behaviour but did not interact with the owner, did not play and/or accept food (Figure 2; step 4 was passed but step 2 was not). Score 2 was assigned to animals that interacted with the owner but not with the experimenter (Figure 2; step 2 was passed but step 3 was not). Animals that interacted (played and/or accepted food) with both the owner and the experimenter obtained a score of 3. Animals with a score less than 3 were re-tested on a different day in the very same environment and experimenter up to a maximum of three times (occasions). The same scoring system was applied on all occasions.

For subjects who were successful on the first occasion, we also measured the latency of passing the test, that is, the time (after the first 5 min of open field) until they started playing with the experimenter or accepted food from her.

### 2.7. Behaviour Variables

The behaviours of the cats and dogs were assessed in terms of posture [36,37], movement (crouching vs. normal moving), and position (close to owner vs. box/blanket). The relative duration (proportion of the time spent by the given behaviour element relative to the duration of the whole test) of these variables was recorded. Originally, some more behaviour variables were coded (i.e., lying, vocalisation, etc.), however, only behaviours that occurred for more than 5% of the total time were included in the analyses (Table 1). The record of four dogs could not be evaluated due to technical reasons.

### 2.8. Data Analysis

We assessed the inter-observer agreements for all behaviours by parallel coding of a random subsample (20% of the videos) calculating the Cohen’s kappa. Intercoder reliability values indicated acceptable–high reliability (mean ± SD: 0.74 ± 0.14, minimum 0.61, maximum 0.92; crouching: 0.76; sitting, standing: 0.74; moving (normal): 0.74; crouching moving: 0.61; close to O: 0.9; close to box/blanket: 0.74).

Percentages of the scores (0–3) received on the first occasion and the proportion of subjects who finally passed vs. did not pass the test from the groups were compared using the Pearson’s chi squared test. A Kendall’s tau-b correlation was run to determine the relationship between the cats’ age and the scores they received on the first occasion. Mann–Whitney U tests were used to compare the behavioural variables of dogs vs. cats and E vs. IE cats and the time the successful subjects needed to pass. Benjamini–Hochberg correction was applied to handle multiple comparisons. Statistical analyses were performed by the SPSS 23.0 statistical software package (SPSS Inc., Chicago, IL, USA).

## 3. Results

### 3.1. Success in Passing the Habituation Test

**Figure 3:**
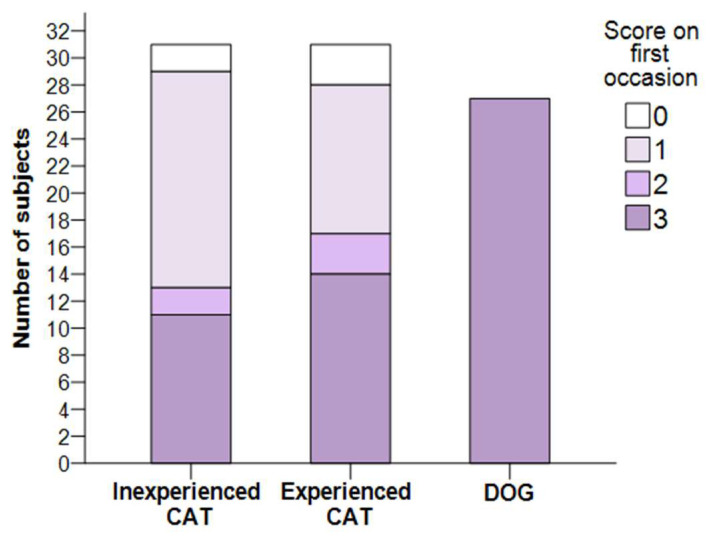
Scores received by dogs and cats (IE and E) on the first occasion in the habituation test. All dogs passed the test on the first occasion.

All dogs passed the habituation test on the first occasion. Of the 62 cats, 25 could be successfully habituated (reached three points) the first time. No statistical analysis was performed to compare the success of the species as all dogs passed the first time. The comparison of the scores of E and IE cats on the first occasion did not show a significant difference (χ^2^(3) = 1.686; *p* = 0.640) (Figure 3).

Of the remaining 37 cats, eight were not brought back for the next occasion because the owners saw no chance of improvement (four/0 points and four/1 point). Of the 29 cats tested the second time, seven passed. Of the 22 remaining cats, eight were not brought back for the third occasion (one/0 point, four/1 point, two/2 points), partly because the owners gave up, partly for other reasons (e.g., illness). Thus, on the third habituation test, a total of 14 cats participated, of which five were successful. In sum, finally 60% of the cats (N = 37) could be successfully habituated.

Experience did not have an effect on the cats’ final success either as 61.3% of E cats and 58.1% of IE cats passed the habituation test (χ^2^(1) = 0.07; *p* = 0.795).

**Figure 4:**
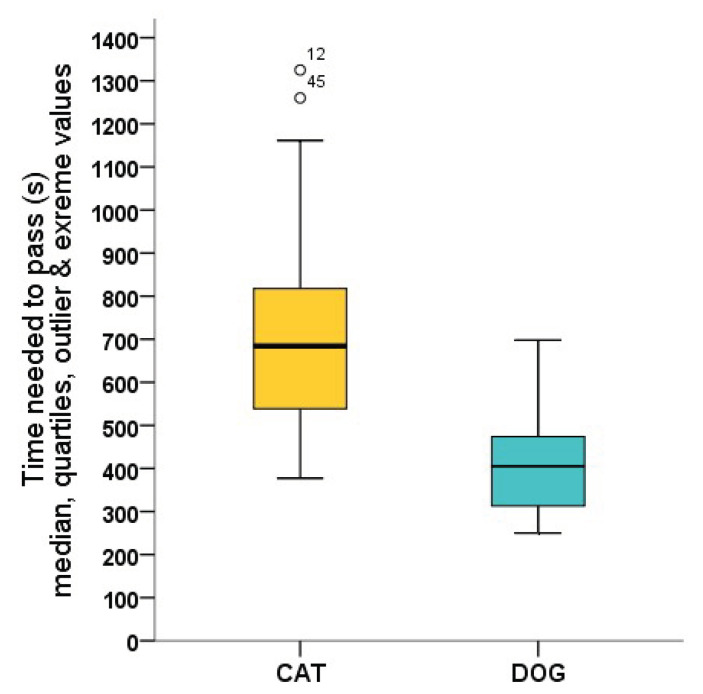
Time (s) needed to pass the test after the 5-min-long open field part. Only data of the cats successfully habituated on the first occasion were analysed.

Even from the individuals that were successful on the first occasion (obtained a score of 3), dogs (N = 27) passed the test significantly faster than cats (N = 25) (U = 64; *p* < 0.001) (Figure 4).

**Figure 5:**
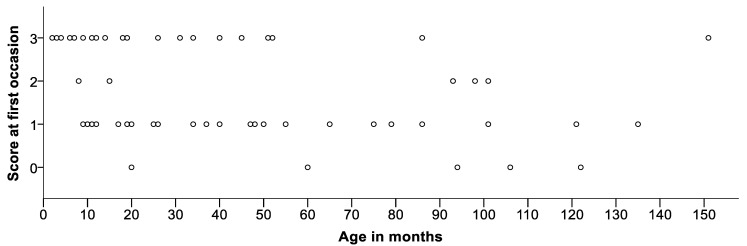
The cats’ scores received on the first occasion of the habituation test based on their age.

In cats, on the first occasion, there was a significant negative correlation between age and success; younger cats obtained higher scores (τ_b_ = −0.38, *p* < 0.001) (Figure 5). As all dogs obtained a score of 3 on the first occasion, this analysis was not performed in dogs.

### 3.2. Behavioural Observations during the First 5 Minute of the First Occasion

**Figure 6:**
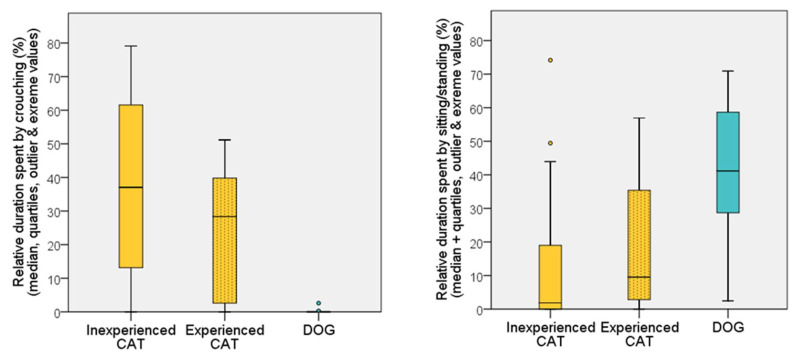
Relative duration (%) of time spent in different postures (crouching—sitting/standing) by dogs and cats during the first 5 min of the habituation test on the first occasion.

We revealed significant differences between the behaviour of dogs and cats in all variables during the first 5 min (see below), but no such differences between E and IE cats were found; all had a *p* > 0.05 with the only exception of sitting/standing (U = 313.5; *p* = 0.017), which was no longer significant after the Benjamini correction was applied. Note that in the figures, we display three groups to illustrate the behaviour of the two cat groups separately, but statistical comparisons for dogs vs. cats were conducted using the data of all cats (N = 62) (as we could not test IE dogs). Compared to dogs, cats spent more time in crouching position (U = 151.5; *p* < 0.001) and spent less time sitting/standing (U = 233.5; *p* < 0.001) (Figure 6).

**Figure 7:**
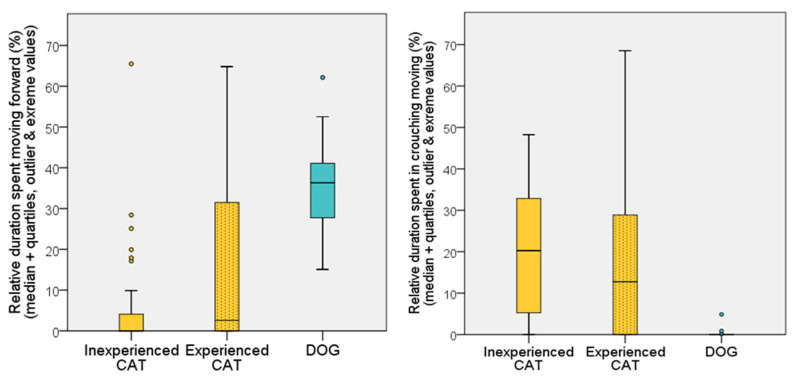
Relative duration (%) of time spent in different movements (forward—crouching) by dogs and cats during the first 5 min of the habituation test on the first occasion.

We found that cats spent more time moving in a crouched position (U = 232.5; *p* < 0.001), while we could observe more (typical) moving forward in the case of dogs (U = 188; *p* < 0.001) (Figure 7).

**Figure 8:**
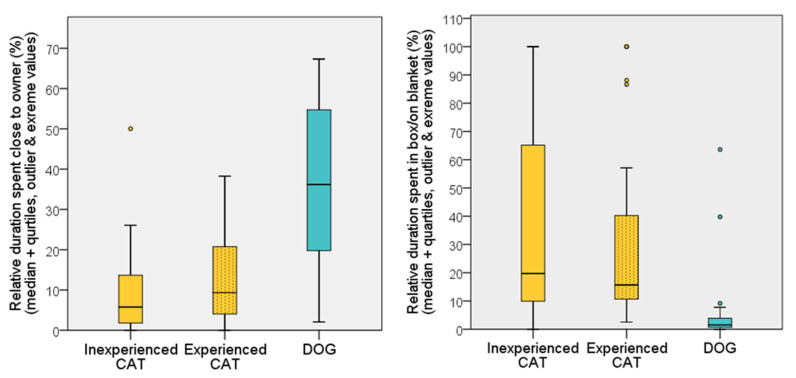
Relative duration (%) of time spent close to the owner and to the box/blanket by dogs and cats during the first 5 min of the habituation test on the first occasion.

Dogs spent more time close to the owner than cats (U = 240; *p* < 0.001), while cats spent more time close to their box than dogs were close to their blanket (U = 179; *p* < 0.001) (Figure 8).

## 4. Discussion

The main aim of this study was to assess whether the remarkable differences between the ecology, domestication process, and the development of cats and dogs and in the individual experiences within cats had an effect on the ability to habituate to a novel environment: an unfamiliar laboratory and a friendly stranger.

Our results showed that during the first five minutes (open-field), dogs and cats appeared to choose different strategies; dogs left their starting location rapidly, and started to explore the novel place (sitting-standing, typical moving forward), while cats stayed much longer in their box, and even after emergence, they typically showed passive avoidance, spending much time by crouching or crouching movements. Crouching has been reported as a typical passive defence response when animals can neither flee from nor find a hiding place [38]. Another typical response of cats in our experiment, hiding in the box or staying close to it, is a well-known stress related behaviour in many species. Latency of leaving the box is often used to assess aversive experiences and long latencies are associated with fear and acute stress [39]. Cats were reported to have spent most of their time in their hiding box during the first two weeks after being housed in a novel environment [40], and shelter cats spent 55% of the time in the hiding box in a novel environment [41]. Thus, similarly to observations in different contexts (e.g., [40,42], we found that novel situations are very stressful for cats, which leads to inactivity and the inhibition of behaviours such as exploration, feeding, or play. There could be several, not mutually excluding factors to explain the observed species differences. This study did not aim to detangle these factors but, for the first time, to directly compare the behaviours of the two species in an environment they are (or supposed to be) tested to compare some of their socio-cognitive skills and behaviour.

The less successful habituation of cats compared to dogs is most likely to be explained by (i) differences in their ecology (for cats, it is less natural to leave their habitat, thus they feel less comfortable in a novel place and they are both a predator and prey species so they could be expected to behave like a prey species) or (ii) the specificity of domestication (cats have been living next to humans and not with them) between the two species. The difference in the experience (keeping conditions) of the two species can either be the result of these ultimate factors or can act as an independent factor. 

The different degree of stress in the two species in the strange environment could be complemented by the effects of their different relationship with the human social partner, that is, the cat–owner relationship may lack the owner’s safe haven effect (in dogs, see [23]). The much greater time dogs spent close to their owners, even though they also spent more time by active exploration, suggests that even if they were scared in the strange place or by the unfamiliar experimenter, the presence of the owner could provide them with security and comfort. In dogs [43] and human infants [44], proximity seeking with the caregiver in a strange situation (unfamiliar place and person) is one of the most important indicators of attachment behaviour. While adult family dogs show human analogous attachment behaviour towards their owner [45] and even the presence of a passive owner alters the dogs’ behaviour in an ambiguous situation [23], the behaviour of companion cats suggest that they do not show similar attachment behaviour towards their owners [29]. Though cats were reported to show preference for the owner after separation in a novel environment [19], based on our results, not the owner but the box seem to represent a ‘safe haven’ for them, while dogs could use the owner as aa ‘secure base’ for exploration.

In addition to the species-specific differences, we expected that cats who could explore novel places and meet strangers regularly would perform more like dogs compared to inexperienced cats. To our surprise, experience neither played a significant role in the cats’ success nor affected their behaviour. We note, however, that even our experienced cat group could have much less opportunity to become used to unfamiliar places and persons than typical family dogs, because even the most experienced cats are rarely exposed to the same amount and variability of stimuli as that of average dogs. Companion dogs often have the chance to explore novel places and meet unfamiliar humans, and they can learn to cope with the extreme plasticity of this social environment [25]. Of note, unlike dogs, outdoor cats face novel stimuli in a mostly familiar environment, and they can avoid such encounters because they know how to escape in that environment. In our test design, however, cats faced a situation that was novel for them in all respects.

We expected that age may have an effect on the cats’ tendency to explore the novel place and interact with an unfamiliar person. Indeed, younger cats proved to be more successful and adapted better to the test situation. Our findings may be related to that novelty seeking in cats is more needed before the age when feral cats establish their own territory [46]. So far, very little is known about the age-related effects of companion cats’ behaviour because socio-cognitive tests have been performed on adults and research on kittens has basically focused on the factors that affect their successful socialisation [47]. Though Lowe and Bradshaw [48] experimentally demonstrated the relative stability of cats’ responses to being handled by an unfamiliar person between 2 and 33 months of age, they tested the cats’ reaction in a rather simple situation (being held for 1 min) where the cats were restrained. Casey and Bradshaw [49] compared the effect of regular and enhanced handling of kittens during the socialisation period, and one year later, the owners of those with the enhanced handling reported fewer signs of fear of humans. As we did not have data about the early socialisation of the subjects in our experiment, we could not consider the potential impact of this factor.

Given the high variance in the cats’ responses, which does not seem to be highly related to differences in experience, we could argue that genetic factors related to boldness and sociability can be involved [50]. Individual personality type may also play a role in both the cats’ first reaction and their ability to habituate over the three sessions, given that this was reported to be a significant factor influencing the behaviour of adult cats when they first encountered unfamiliar persons in a laboratory setting [51]. Further investigation of the difference between cat breeds [52,53] and cats that do not belong to any breed (house cats) could also provide better insights into the background of the cats’ adaptability to a novel environment.

## 5. Conclusions

In summary, a comparison of the results of dogs and cats when two to three times as many cats are required for the same number of successfully tested subjects cannot be considered as a valid analysis at the group level. From a broader perspective, considering the growing number of comparative studies [54] where the validity of the species comparison can be compromised by either different or exactly the same test procedures, to figure out to what extent lab tests are feasible for a species is of crucial importance. Our findings have major implications in collecting reliable and repeatable data on cat behaviours and in ensuring optimal welfare, relevant not only in laboratory, but also in other settings (e.g., vet clinics).

## Figures and Tables

**Table 1:** Description of the behaviours observed in both cats and dogs.

Posture	Crouching	The body is close to the ground, all four legs are bent, and the belly is close to the ground, muscle is tensed—without moving.
	Sitting, Standing	Upright and immobile position, with the hind legs flexed, resting on the ground and the front legs extended straight or with all four paws on the ground and legs extended, supporting the body.
Movement	Moving (normal)	Forward locomotion in an upright (not crouched) position
	Crouching moving	Slow, forward locomotion in a crouched position
Position	Close to O	The head of the pet is in reaching distance (within 30 cm) to O
	Close to box/blanket	The head of the pet is within 30 cm to the box/blanket (including being inside the box/on the blanket)

## Data Availability

The data presented in this study are available on request from the corresponding author. The data are not publicly available due to ongoing further analysis.
